# *Pseudomonas* 1-Aminocyclopropane-1-carboxylate (ACC) Deaminase and Its Role in Beneficial Plant-Microbe Interactions

**DOI:** 10.3390/microorganisms9122467

**Published:** 2021-11-29

**Authors:** Bernard R. Glick, Francisco X. Nascimento

**Affiliations:** 1 Department of Biology, University of Waterloo, Waterloo, ON N2L 3G1, Canada; glick@uwaterloo.ca; 2 iBET, Instituto de Biologia Experimental e Tecnológica, Apartado 12, 2781-901 Oeiras, Portugal

**Keywords:** *Pseudomonas*, 1-aminocyclopropane-1-carboxylic acid, ethylene, plant-microbe interactions

## Abstract

The expression of the enzyme 1-aminocylopropane-1-carboxylate (ACC) deaminase, and the consequent modulation of plant ACC and ethylene concentrations, is one of the most important features of plant-associated bacteria. By decreasing plant ACC and ethylene concentrations, ACC deaminase-producing bacteria can overcome some of the deleterious effects of inhibitory levels of ACC and ethylene in various aspects of plant-microbe interactions, as well as plant growth and development (especially under stressful conditions). As a result, the *acdS* gene, encoding ACC deaminase, is often prevalent and positively selected in the microbiome of plants. Several members of the genus *Pseudomonas* are widely prevalent in the microbiome of plants worldwide. Due to its adaptation to a plant-associated lifestyle many *Pseudomonas* strains are of great interest for the development of novel sustainable agricultural and biotechnological solutions, especially those presenting ACC deaminase activity. This manuscript discusses several aspects of ACC deaminase and its role in the increased plant growth promotion, plant protection against abiotic and biotic stress and promotion of the rhizobial nodulation process by *Pseudomonas.* Knowledge regarding the properties and actions of ACC deaminase-producing *Pseudomonas* is key for a better understanding of plant-microbe interactions and the selection of highly effective strains for various applications in agriculture and biotechnology.

## 1. Introduction

The unacceptable levels of pollution and other negative environmental impacts caused using chemical fertilizers and pesticides in agriculture is a major threat to food/soil security and overall human and animal health. Hence, achieving sustainable and efficient agricultural practices is one of the major challenges of this century.

The direct application of plant-growth-promoting bacteria (PGPB), beneficial members of the plant and soil microbiome, is a powerful alternative to the use of polluting chemical compounds [1]. These bacteria may facilitate plant growth, development and stress resistance through a wide range of mechanisms, including the manipulation of plant hormone levels [2].

*Pseudomonas* is a highly diverse bacterial genus that currently encompasses more than 250 species, including common members of the known plant and soil microbiomes worldwide and several PGPB. Because of their increased metabolic versatility, fast growth rate, biocontrol activities, ability to survive in a variety of soils and to directly interact with plant hosts, several *Pseudomonas* strains are of particular interest for the development of products for agricultural and biotechnological applications. Nevertheless, the selection of highly effective PGP *Pseudomonas* strains is still a challenge.

One way to address this challenge resides with the selection of *Pseudomonas* strains presenting the ability to manipulate plant hormone concentrations, specifically the gaseous plant hormone ethylene, which is an important regulator of multiple aspects of plant development as well as stress resistance and plant-microbe interactions [3,4]. In this regard, the expression of 1-aminocyclopropane-1-carboxylate (ACC) deaminase, an enzyme that is responsible for the cleavage of the non-proteinogenic amino acid ACC [5], the immediate precursor of ethylene in all higher plants, plays a key role in the bacterial ability to modulate plant ethylene levels [6]. The capacity to consume plant ACC has been demonstrated to increase the PGP abilities of numerous bacterial strains, including *Pseudomonas* spp. Importantly, several studies have revealed that the *acdS* gene encoding the ACC deaminase enzyme is highly prevalent and positively selected in bacteria that closely associate with plants [7], including a large number of rhizobial symbionts (those belonging to both the Alpha and Betaproteobacteria classes) [8]. Moreover, The microbiome of plants that normally grow under stress conditions is typically enriched in *acdS*-containing bacteria, including *Pseudomona*s [9,10,11].

In this work, several aspects of *Pseudomonas* ACC deaminase production and its important role in plant-microbe interactions are reviewed in detail.

## 2. *Pseudomonas*: Common and Important Members of the Plant Microbiome

Bacteria belonging to the genus *Pseudomonas* are commonly found worldwide in soils and in close association with plants. This diverse group of bacteria (polyphyletic) [12] can be found in the plant rhizosphere (the portion of soil directly associated with the plant roots) [13,14,15], but also colonizing internal plant tissues (acting as endophytes) [16,17], external plant tissues such as shoots and leaves (acting as epiphytes) [18,19], as well as some specialized plant organs like leguminous plant root nodules [20,21,22]. Soil- and plant-associated *Pseudomonas* strains may also present different ecological roles, ranging from beneficial actions to pathogenicity (e.g., *P. syringae* group species) [23].

Beneficial *Pseudomonas* play key roles in soil nutrient cycles (N, P, K, C), soil health via the catabolism of several deleterious compounds (e.g., heavy metals and aromatic compounds) and the suppression of several pathogens by producing a wide range of antimicrobial compounds such as lipopeptides and antibiotics [24,25,26,27]. When associated with plants, *Pseudomonas* strains may potentiate its host’s growth by facilitating nutrient acquisition (P, N, K, Fe) [28,29,30,31], or by the modulation of plant hormone concentrations (e.g., indoleacetic acid (IAA) biosynthesis and catabolism, biosynthesis of cytokinins, catabolism of ACC) [32,33,34,35]. Moreover, several *Pseudomonas* strains activate plant defense responses and induce systemic resistance through the activation of specific plant signaling mechanisms via their Microbe-Associated Molecular Patterns (MAMPs), effectors and other synthesized compounds [36].

## 3. Ethylene and ACC: Master Regulators of Plant Growth, Development, and Plant-Microbe Interactions

Ethylene (C_2_H_4_) is a gaseous plant hormone that is synthesized in plants via an ACC-dependent pathway in which methionine and *S*-adenosyl-l-Methionine (SAM) are the major precursors (Yang cycle) [37]. Plant SAM is converted to ACC by the enzyme ACC synthase, and then, ACC may be directly converted to ethylene by the enzyme ACC oxidase. These represent the key and limiting steps in ACC and ethylene biosynthesis. Additionally, ACC may be conjugated to other forms such as M-ACC (malonyl-ACC), G-ACC (glutamyl-ACC) and J-ACC (jasmonoyl-ACC) that can be accumulated and transported within plant tissues [3].

Ethylene regulates several aspects of plant growth and development, such as root and shoot elongation, leaf growth, flowering, fruit development and ripening, and root nodule development [38]. Ethylene also regulates the direct responses to biotic and abiotic stresses [4], as well as general plant-bacterial interactions [3], including the symbiotic nodulation process [39].

While ACC was first thought to act only as the ethylene precursor, recent studies have revealed that the role of this non-proteinogenic alpha amino acid is more important than previously thought. These studies showed that ACC may regulate several processes of plant development (e.g., cell division, root elongation) and act as a signaling molecule, independently from ethylene [40,41,42,43,44,45,46,47]. Importantly, Tsang and colleagues [41] showed that bacterial flagellin, a major MAMP and a known inducer of the plant defense response, activated an ACC-dependent and ethylene-independent mechanism involved in the regulation of root elongation. This result indicates that ACC plays a significant role in plant-microbe interactions. Since plant ACC can be transported and exuded [48,49,50], it may also play a significant role as a signaling molecule in the rhizosphere and phyllosphere [3].

## 4. Bacterial ACC Deaminase and the Manipulation of Plant ACC and Ethylene Levels

**Figure 1:**
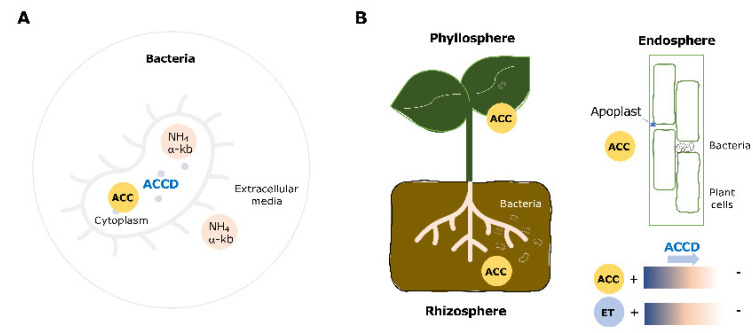
Schematic representation of (**A**) bacterial 1-aminocylopropane-1-carboxylate (ACC) deaminase activity and (**B**) the modulation of plant ACC and ethylene concentrations. ACC-1-aminocyclopropane-1-carboxylate; ET-ethylene; ACCD-ACC deaminase.

The ACC deaminase enzyme directly cleaves ACC, resulting in its conversion into ammonia and alpha-ketobutyrate [5] that can then be used as sole N and C sources by plant-associated bacteria (Figure 1A). ACC deaminase, encoded by the *acdS* gene, is a multimeric enzyme with a subunit molecular mass ranging from 36–42 KDa that is mostly prevalent within soil and plant-associated Proteobacteria (Alpha, Beta, Gamma) and Actinobacteria [7], although it is also found in several other types of bacteria and some fungi. This enzyme is located in the bacterial cytoplasm (i.e., it is not secreted) [51], which is consistent with the lack of transmembrane and/or signal peptides in its sequence, as well as the data regarding its optimal functioning conditions (pH 7–8, the cytoplasmic pH).

Importantly, ACC deaminase-producing bacteria (rhizospheric, endophytic, or epiphytic) can modulate the concentrations of ACC (i) in the rhizosphere and phyllosphere by consuming plant’s exuded ACC, or (ii) within plant tissues (e.g., the endosphere and root nodules), thus, directly limiting the actions of ACC, and subsequently limiting the production of ethylene by the plant host (Figure 1B).

The production of ACC deaminase by plant-associated bacteria and the consequent decrease of plant ACC and ethylene levels results in increased (i) bacterial colonization/competitiveness [52,53], (ii) bacterial nodulation abilities [54,55,56], (iii) plant growth promotion [57,58,59], and (iv) plant tolerance to biotic and abiotic stress [17,60,61,62,63]. Due to its significant impact in plant-microbe interactions, the *acdS* gene is positively selected in plant-associated bacteria including many rhizobial symbionts [8]. For instance, the *acdS* gene was detected in 234 of 395 NodC-containing rhizobia (Alpha and Betaproteobacteria), and in many of these strains the *acdS* gene is maintained in transmissible symbiotic islands and plasmids that also contain the nod (nodulation) and nif (nitrogen fixation) genes [8]. Moreover, the prevalence of *acdS* genes in rhizobial populations is connected to their ability to nodulate specific leguminous plant hosts, suggesting that the plant host plays a role in the selection of ACC/ethylene-modulating genes.

The *acdS* gene is also highly prevalent in the microbial communities of plants subjected to stress conditions [9,10,11]. For example, the abundance of ACC deaminase-producing bacteria was significantly increased in the rhizosphere of wild barley growing under stressful conditions when compared to wild barley grown under non-stressful conditions [9]. Similarly, the presence of ACC deaminase-producing bacteria was increased in the Brassica napus (canola) rhizosphere when the plant was cultivated in a heavy metal contaminated soil [10]. Moreover, the seeds of Arabidopsis thaliana exposed to cadmium for several generations contained more ACC deaminase-producing bacteria, including *Pseudomonas* spp., than the seeds of plants that were never exposed to cadmium stress [11].

## 5. Insights into the Prevalence and Evolution of ACC Deaminase in the Genus *Pseudomonas*

The ACC deaminase gene is present in 2591 *Pseudomonas* genomes (accessed in August 2021), including 39 *Pseudomonas* type strain genomes (Table 1). ACC deaminase was mostly detected in members of the following *Pseudomonas* genomic groups/subgroups (previously determined in Girard et al. [64]): ***P. syringae* group** (*P. syringae*, *P. amygdali*, *P. avellanae*, *P. asturiensis*, *P. cannabina*, *P. capsici*, *P. caricapapayae*, *P. caspiana*, *P. cichorii*, *P. congelans*, *P. ficuserectae*, *P. floridensis*, *P. foliumensis*, *P. tremae*, *P. triticumensis*, *P. viridiflava*); ***P. fluorescens*** **subgroup** (*P. grimontii*, *P. marginalis*, *P. palleroniana*, *P. panacis*), ***P. corrugata subgroup*** (*P. bijieensis*, *P. brassicacearum*, *P. kilonensis*, *P. ogarae*, *P. tehranensis*, *P. thivervalensis*, *P. viciae*; *P. zarinae*); ***P. mandelii* subgroup** (*P. farris*, *P. migulae*); ***P. massiliensis* group** (*P. typographi*); ***P. gessardii* group** (*P. gessardii*); ***P. asplenii* subgroup** (*P. fuscovaginae*); ***P. straminea* group** (*P. flavescens*); ***P. anguilliseptica* group** (*P. benzenivorans*); ***P. oryzihabitans* group** (*P. oryzihabitans*, *P. psychrotolerans*, *P. rhizoryzae*).

**Figure 2:**
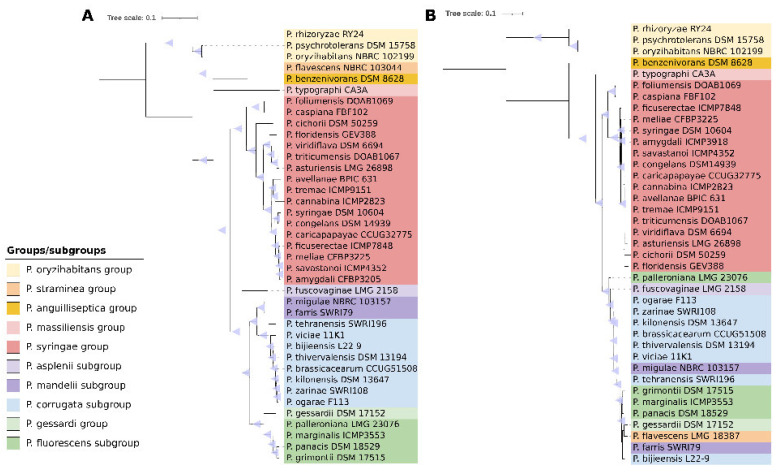
(**A**) Phylogram based on 576 core genes protein sequences from *Pseudomonas* type strains that possess *acdS* genes. The core genes (single copy genes found in all tested strains) were selected based on GHOSTKOALA functional annotation [66] and a python script built in house. The core genes were individually aligned using MAFFT [67] and concatenated using a python script built in house. The phylogenetic analysis was conducted in GalaxyPasteur server [68] using FastTree v2.1.10 [69], the LG model and a bootstrap of 100 replications. (**B**) Phylogram based on the AcdS sequences of *Pseudomonas* type strains. The sequences were obtained from the NCBI database and aligned using MAFFT [67]. The phylogenetic analysis was conducted in GalaxyPasteur server [68] using FastTree v2.1.10 [69], the LG model and a bootstrap of 1000 replications.

Overall, the majority of the AcdS-containing *Pseudomonas* strains clustered in specific *Pseudomonas* groups/subgroups (Figure 2A) and most of *acdS* genes presented similar GC% when compared to the strain overall genome GC% (Table 1), indicating that the presence and evolution of ACC deaminase is mostly linked to the overall strain’s genomic properties/evolutionary history [7]. Nevertheless, the phylogenetic and comparative analysis based on 576 core genes (Figure 2A) and AcdS (Figure 2B) sequences showed that *AcdS* evolution in some *Pseudomonas* clades is difficult to resolve. The data suggests that some clades (e.g., *P. fluorescens* group) have possibly acquired *acdS* genes via past horizontal gene transfer (HGT) or recombination events between *Pseudomonas* strains that occurred in a more recent time. For example, members of the *P. fluorescens* subgroup could be easily distinguished based on their core genes (576 protein sequences) (Figure 2A), however, its AcdS sequences were highly similar to those of members of the *P. corrugata* and *P. mandelli* subgroups (Figure 2B). Alternatively, the *acdS* gene is less prone to modifications in these *Pseudomonas* subgroups.

*Pseudomonas* flavescens LMG 18387^T^ and *P. fuscovaginae* ICMP 5940^T^ seem to have indeed acquired *acdS* genes via HGT from other members of the *Pseudomonas* genus. The analysis also suggested that some *Pseudomonas* may have acquired *acdS* genes through more distant HGT events (between less related strains). This seems to be the case for *P. benzenivorans* DSM 8628^T^, which possesses an *acdS* gene presenting a GC% of 55.4 despite presenting an overall genomic GC% of 65.2. The phylogram based on AcdS sequences (Figure 2B) showed that *P. benzenivorans* DSM 8628^T^ *AcdS* formed a unique cluster. BLASTp analysis revealed that the AcdS sequence from *P. benzenivorans* DSM 8628^T^ was mostly similar (~85% identity) to the AcdS of *Alphaproteobacteria*, namely, *Bosea*, *Methylobacterium*, and *Bradyrhizobium*. These results suggest that *P. benzenivorans* DSM 8628^T^, possibly acquired an *acdS* gene from an *Alphaproteobacteria* donor via HGT.

The phylogram based on AcdS sequences (Figure 2B) also demonstrated that members of the *P. oryzihabitans* group present a different ACC deaminase compared to other *Pseudomonas* (~56% identity) (Figure 2B). In a previous report, Nascimento et al. [7] observed that members of the *P. oryzihabitans* group formed a unique AcdS cluster, distantly from the AcdS of other *Proteobacteria*. At this point it is difficult to ascertain the true evolutionary history of the *P. oryzihabitans* group AcdS. However, members of the *P. oryzihabitans* group possess high GC% genomes (~65%) compared to other *Pseudomonas* (58–61% GC content) (Table 1) and the *acdS* genes of these strains also present a high GC% content and are shorter (1014 bp) (Table 1). These data suggest that either (i) the adaptation to a high GC% genome impacted the *P. oryzihabitans* group ACC deaminase evolution; or (ii) the high GC% *P. oryzihabitans* group acquired a high GC% *acdS* gene from an unknown donor. New studies are necessary to understand the evolution of ACC deaminase in the *P. oryzihabitans* group.

Interestingly, *Pseudomonas* AcdS^+^ groups/clades are predominantly composed of plant-associated *Pseudomonas* (both PGPB and plant pathogens) (Table 1), which is consistent with previous studies reporting the increased prevalence of *acdS* genes in plant-associated bacteria [7,8]. The acquisition and/or maintenance of *acdS* genes seems to be favored in specific but not all plant-associated *Pseudomonas*. The factors regulating this selection remain to be determined.

Ultimately, due to its increased prevalence in both PGP and plant pathogenic *Pseudomonas* (e.g., *P. syringae* group), the mere presence of *acdS* genes cannot be used to predict beneficial interactions with a plant host.

## 6. *Pseudomonas* ACC Deaminase

The *Pseudomonas* ACC deaminase protein sequences present some variability, which is consistent with the different distribution patterns of *acdS* genes in the different *Pseudomonas* groups (Figure 2B). Alignments showed that *Pseudomonas* AcdS sequences are somewhat conserved, presenting 37.8% identical sites. These include the Lys51, Ser78, Tyr295, Glu296 and Leu322 residues that are necessary for ACC deaminase activity [70]. The *Pseudomonas acdS* genes present similar sizes (1014–1017 bp), and, consequently, generate similar proteins with a predicted subunit weight of ~36.6 KDa.

The ACC deaminase enzyme of *Pseudomonas* sp. GR12-2 [51] and *Pseudomonas* sp. UW4 have been characterized in some detail [65]. The enzyme of strain GR12-2 was found in the bacterial cytoplasm, presented a subunit molecular mass of 35 kDa and its activity was optimal at 30 °C and a pH optimum of 8.5 [51]. The *Pseudomonas* sp. UW4 ACC deaminase presented a molecular weight of ~41 kDa and showed a K_M_ = 3.4 ± 0.2 mM and k_cat_ = 146 ± 5 min^−1^ at pH 8.0 and 22 °C. The strain UW4 enzyme was thermodynamically stable presenting a melting temperature of 58 ± 1 °C. The *Pseudomonas* sp. UW4 ACC deaminase K_M_ and k_cat_ values are comparable to those of other ACC deaminase-producing bacteria (Table 2), including *Methylobacterium* strains [71] and *Amycolatopsis methanolica* 239 [72]. However, the enzyme of *Pseudomonas* sp. UW4 presented a higher K_M_ (indicating less tight binding of the substrate ACC) and an increased k_cat_ and presented a different temperature optimum (37 °C) when compared to the ACC deaminase from these other strains.

**Figure 3:**
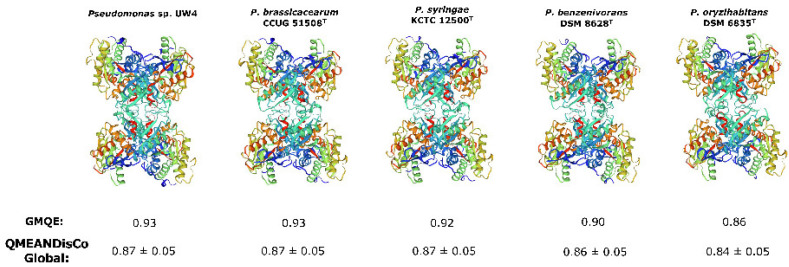
Models of the different ACC deaminase enzymes from selected *Pseudomonas*. The models were created in Swiss-Model [73] using the crystal structure of ACC deaminase from *Burkholderia* (formerly *Pseudomonas*) sp. ACP as template (SMTL ID: 1tzm.1) [74]. GMQE—Global Model Quality Estimate; QMEANDisCo is a composite score for single model quality estimation.

Several studies have suggested that the ACC deaminase from bacterial strains form homotetramers (Table 2). The modeling of different *Pseudomonas* AcdS (Figure 3), including those of *P. benzenivorans* DSM 8628^T^ and *P. oryzihabitans* DSM 6835^T^, revealed that this conformation is favored. The obtained AcdS structural models presented increased values of overall quality (Figure 3), indicating structural conservation amongst *Pseudomonas* ACC deaminases.

## 7. The Role of ACC Deaminase in Beneficial *Pseudomonas* Plant Growth Promotion and Plant Protection Abilities

Several authors have obtained *acdS* mutants (including both loss and gain of function) of different beneficial *Pseudomonas* strains in an effort to understand the direct role of ACC deaminase in *Pseudomonas* plant growth promotion and plant protection abilities. These studies have revealed that the expression of ACC deaminase greatly impacts the performance of the different *Pseudomonas* strains, and clearly regulates their ability to modify several aspects of plant growth and development (Table 3).

### 7.1. Root Development Induced by Pseudomonas

The loss of the ability to promote plant root length development is one of the most described effects in *Pseudomonas* ACC deaminase minus mutants (loss of function) (Table 3). For instance, the *acdS^−^* mutants of *Pseudomonas* sp. GR12-2, *Pseudomonas* sp. UW4, *P. brassicacearum* YsS6 (formerly *P. fluorescens*) and *P. migulae* 8R6 all lost the ability to promote canola root elongation [17,35]. The *acdS^−^* mutant of *P. ogarae* F113 (formerly *P. kilonensis*) did not promote root length and root numbers in the maize cultivar EP1 [75]. On the other hand, *P. protegens* CHA0, expressing an exogenous ACC deaminase gene, gained the ability to promote canola root elongation [76], and, *P. putida* ATCC 17399 containing the broad-host-range plasmid pRKACC and expressing an exogenous ACC deaminase gene, gained the ability to promote root length development and adventitious root formation in tomato plants subjected to flooding [50].

### 7.2. Delay in Flower Senescence by Endophytic Pseudomonas

The inoculation of mini-carnation cut flowers with the ACC deaminase-producing endophytes, *P. brassicacearum* YsS6 and *P. migulae* 8R6, resulted in a delay in flower senescence of several days, and, consequently, an increased flower shelf-life [72]. These effects were not observed when the plants were inoculated with the respective *Pseudomonas acdS*^−^ mutants of these strains [77]. Interestingly, the ACC deaminase-producing endophytes provided 2 additional days of shelf-life compared to the application of 1-aminoethoxyvinylglycine (AVG), a chemical compound known to limit the biosynthesis of ethylene. Moreover, the incubation of cut flowers with *Pseudomonas* sp. UW4, a rhizospheric strain (unable to colonize the plant interior) did not affect the senescence of cut flowers. This data clearly indicates that the use of *Pseudomonas endophytes* with ACC deaminase activity has the potential to replace the chemicals that are currently used by the cut flower industry to increase the life of cut flowers.

### 7.3. Plant Protection against Abiotic Stress

*Pseudomonas acdS*^−^ mutant strains are greatly impaired in their ability to protect plants from abiotic stress (Table 3). For example, the *Pseudomonas* sp. UW4 *acdS*^−^ mutant presented a decreased ability to protect canola from salt stress. Cheng and colleagues [61] observed that in the presence of 150 mmol/L salt, canola plants inoculated with wild-type *Pseudomonas* sp. UW4 presented similar biomass values compared to plants grown with no salt added. On the other hand, plants inoculated with the *Pseudomonas* sp. UW4 *acdS^−^* mutant only accumulated approximately 65% of the amount of biomass observed in the absence of salt [61]. Similarly, the *Pseudomonas* sp. UW4 *acdS^−^* mutant also presented a decreased ability to promote cucumber [79] and tomato [81] salt stress resistance compared to its wild-type counterpart. The endophytes, *P. brassicacearum* YsS6 and *P. migulae* 8R6, both presenting ACC deaminase activity, not only promoted tomato plant growth in the absence of stress conditions but also induced the accumulation of much higher fresh and dry biomass, higher chlorophyll contents, and a greater number of flowers and buds in tomato plants subjected to salt stress compared to non-inoculated plants or those inoculated with the respective *Pseudomonas acdS*^−^ mutants [17]. Recently, Liu and colleagues [85] observed that the inoculation of tomato plants with the ACC deaminase-producing *P. azotoformans* CHB 1107 resulted in increasing plant shoot and root dry weights and a significant reduction in the plant ethylene emission in response to salt stress. These beneficial effects were lost when plants were inoculated with *P. azotoformans* CHB 1107 M (*acdS^−^* mutant). Moreover, *P. azotoformans* CHB 1107 significantly increased plant K, Ca, and Mn uptake compared with *P. azotoformans* CHB 1107 *acdS^−^* mutant [85].

Root inoculation of *Rumex palustris* and *Arabidopsis thaliana* plants with ACC deaminase-producing *Pseudomonas* sp. UW4 significantly decreased the shoot cadmium concentration and total content compared to the *Pseudomonas* sp. UW4 ACC deaminase-deficient mutant (*acdS*^−^) inoculation and the non-inoculated control [87]. The *Pseudomonas* sp. UW4 ability to decrease heavy metal accumulation in some plant tissues was correlated with its capacity to express ACC deaminase and decrease plant ethylene levels [87].

The expression of exogenous ACC deaminase genes in *Pseudomonas* also leads to their increased ability to protect plants from other abiotic stresses. Tomato plants inoculated with *P. putida* ATCC 17399 pRKACC, expressing ACC deaminase, had an increased ability to tolerate flooding stress [50]. Similarly, the inoculation of *P. frederiksbergensis* OS211-*acdS* (carrying the pRKACC plasmid and expressing ACC deaminase), resulted in a reduced ethylene emission, less ACC accumulation and lower ACC oxidase activity (52%, 75.9% and 23.2%, respectively) in tomato plants subjected to chilling stress (compared to non-inoculated plants) [86]. The transformed strain, *P. frederiksbergensis* OS211-*acdS*, showed a better plant growth promotion/protection performance when compared to the wild-type strain that lacked ACC deaminase [86], clearly demonstrating the beneficial effect of ACC deaminase expression.

### 7.4. Plant Protection against Biotic Stress

Several ACC deaminase-producing *Pseudomonas* strains, but not their *acdS*^−^ mutants or wild-type counterparts that do not express *acdS*, demonstrated an increased capacity to protect plants from stress induced by pathogens (Table 3). The *Pseudomonas* sp. UW4 *acdS^−^* mutant showed a decreased ability to suppress tomato crown gall development induced by Agrobacterium, compared to the *Pseudomonas* sp. UW4 wild-type strain [63,88]. Moreover, the level of ethylene per mass of internodes carrying Agrobacterium-induced galls was significantly lower in plants pretreated with *Pseudomonas* sp. UW4, than in plants pretreated with the *Pseudomonas* sp. UW4 *acdS^−^* mutant [63].

The inoculation of pine seedlings with the ACC deaminase-producing *Pseudomonas* sp. UW4 led to a significant increase in the plant’s ability to resist the infection caused by the pinewood nematode, *Bursaphelenchus xylophilus*, and reduced the development of pine wilt disease symptoms [60]. This result was not observed when the pine seedlings were inoculated with the *Pseudomonas* sp. UW4 *acdS^−^* mutant [60].

The expression of ACC deaminase by the endophyte, *P. migulae* 8R6, played a key role this bacterium’s ability to improve the resistance of *Catharantus roseus* (*Madagascar Periwinkle*) to phytoplasma infection [62]. On the other hand, the *P. migulae* 8R6 *acdS^−^* mutant was not able to significantly reduce the disease symptoms [62].

### 7.5. Promotion of the Rhizobial Nodulation Process

The expression of ACC deaminase by *P. brassicacearum* YsS6 played a significant role in its ability to promote the nodulation process of both alpha (*Rhizobium tropici* CIAT 899) and beta-rhizobia (*Cupriavidus taiwanensis* STM 894), and, ultimately, leguminous plant growth [82]. The co-inoculation of rhizobial strains with the *P. brassicacearum* YsS6 *acdS^−^* mutant did not result in any increase in the rhizobial nodulation or growth of legume plants [82]. In addition, it was recently shown that *Pseudomonas* sp. Q1 (*P. palleroniana*), but not its *acdS^−^* mutant, promoted the symbiotic performance of *R. leguminosarum bv. trifolii* ATCC 14480^T^ and *Ensifer meliloti* ATCC 9930^T^ under both normal and excess Mn stress conditions [84].

## 8. ACC-Deaminase-Producing *Pseudomonas* and Their Potential Application in the Field

A formulation containing the ACC deaminase-producing *P. fluorescens* TDK1 was developed and applied in two consecutive field trials in saline soils, resulting in the increase of groundnut plant height, number of pods per plant, pod filling per cent and 100 seed weigh [89]. The *P. fluorescens* TDK1 strain greatly improved groundnut growth and saline stress resistance compared to the non-inoculated control and other formulations containing *Pseudomonas* that did not presented ACC deaminase activity [89].

The use of a bacterial consortia composed of ACC deaminase-producing *P. palleroniana* DPB13, *P. palleroniana* DPB16, *Pseudomonas* sp. DPB15 and *Ochrobactrum anthropi* DPC9 significantly increased several key parameters of rice and wheat growth and nutrient content [90]. For example, the inoculated rice and wheat plants significantly increased their nitrogen, phosphorus, potassium, calcium, and sodium contents. The treated plants also increased their 1000 grain weight (10.2%, 40.7%; rice and wheat, respectively), number of grains per panicle/spike (45.5%, 60.6%, rice and wheat respectively), and tillers (32.2%, 106.6%, rice and wheat respectively)[90].

The application of a bacterial inoculant based on four ACC deaminase-producing *Pseudomonas* strains improved sweet corn (*Zea mays* L. var. saccharata) productivity under the limited availability of irrigation water [91]. The study demonstrated that the combination of *Pseudomonas* sp. strains P1, P3, P8, and P14 significantly increased the ear and canned seed yield of sweet corn (44%, and 27%, respectively) compared to the non-inoculated control [91].

## 9. Conclusions and Future Perspectives

Beneficial plant-associated *Pseudomonas* containing ACC deaminase are of great interest for the study of plant-microbe interactions and for the development of novel inoculants for agricultural and biotechnological applications, especially those subjected to stress conditions. These unique strains have evolved in close association with plants worldwide and this has led to an important bacterial adaption to the soil/plant environment. The plant-associated lifestyle may be vital for the increased success of these strains as commercial bacterial inoculants, and ultimately, the most relevant factor for differentiating ACC deaminase-producing *Pseudomonas* groups from other pseudomonads.

Numerous other *Pseudomonas* species that are commonly found in the rhizosphere and associated with plants do not possess *acdS* genes (only 39 strains from hundreds of type strains possess *acdS* genes). This raises some questions:

- What are the factors regulating the selection of ACC deaminase genes in specific Pseudomonas groups (including many Pseudomonas plant pathogens)?
- Is the presence of ACC deaminase linked to a specific Pseudomonas lifestyle/mode of action (e.g., strong plant colonization and activation of plant defense responses)?
- Which genes were co-selected and co-evolved with ACC deaminase genes in the genomes of beneficial and pathogenic plant-associated Pseudomonas?
- If ACC deaminase-producing Pseudomonas strains are key players in promoting plant growth and stress resistance, what is their impact in the overall plant microbiome assembly?
- Which beneficial ACC deaminase-producing Pseudomonas groups could be selected for future field applications worldwide?

Future studies will be necessary to address these questions and gain additional insight into the genomics and physiology of ACC deaminase-producing *Pseudomonas*.

## Figures and Tables

**Table 1:** Properties of ACC deaminase-containing *Pseudomonas* type strains.

*Pseudomonas* Groups	Type Strain	Isolation Source	Isolation Country	Genome GC%	*acdS* GC%	*acdS* length (bp)
*P. syringae* group	*P. syringae* KCTC 12500	*Syringa vulgaris*	Great Britain	58.9	61.3	1017
	*P. amygdali* ICMP 3918	*Prunus amygdalus*	Greece	58.2	59.7	1017
	*P. avellanae* JCM 11937	*Corylus avellata*	Greece	58.5	61.4	1017
	*P. asturiensis* DSM 100247	*Glycine max*	Spain	59.1	61.8	1017
	*P. cannabina* DSM 16822	*Cannabis sativa*	Hungary	58.5	60.8	1017
	*P. capsici* Pc19-1	*Capsicum annuum*	USA	58.4	60.4	1017
	*P. caricapapayae* CCUG 32775	*Carica papaya*	Brazil	58.3	59.8	1017
	*P. caspiana* FBF102	*Citrus*	Iran	57.0	57.6	1017
	*P. cichorii* DSM 50259	*Cichorium endivia*	Germany	58.1	59.8	1017
	*P. congelans* DSM 14939	Phyllosphere of grasses	Germany	59.3	61.7	1017
	*P. ficuserectae* ICMP 7848	*Ficus erecta*	Japan	57.9	60	1017
	*P. floridensis* GEV388	Tomato	USA	59.2	60.1	1017
	*P. foliumensis* DOAB 1069	Wheat phyllosphere	Canada	57.2	57.9	1017
	*P. meliae* CFBP 3225	*Melia azedarach*	Japan	58.4	59.4	1017
	*P. savastanoi* ICMP4352	*Olea europaea*	Yugoslavia	58.0	59.7	1017
	*P. viridiflava* DSM 6694	Dwarf or runner bean	Switzerland	59.4	62.0	1017
	*P. tremae* DSM 16744	*Trema orientalis*	Japan	57.8	59.3	1017
	*P. triticumensis* DOAB 1067	Wheat phyllosphere	Canada	59.3	61.2	1017
*P. corrugata* subgroup	*P. brassicacearum* CCUG 51508	Rhizoplane of *Brassica napus*	France	60.8	57.3	1017
	*P. bijieensis* L22-9	Cornfield soil	China	60.9	60	1017
	*P. kilonensis* DSM 13647	Agricultural soil	Germany	60.9	58.3	1017
	*P. thivervalensis* DSM 13194	Rhizoplane of *Brassica napus*	France	61.2	58.4	1017
	*P. viciae* 11K1	Rhizosphere broad bean	China	60.3	59.8	1017
	*P. tehranensis* SWRI196	Rhizosphere of wheat	Iran	60.5	61.4	1017
*P. straminea* group	*P. flavescens* LMG 18387	Walnut tree, canker tissue	USA	63.5	60.1	1017
*P. asplenii* subgroup	*P. fuscovaginae* ICMP 5940	*Oryza sativa*	Japan	61.4	56.6	1017
*P. gessardii* subgroup	*P. gessardii* DSM 17152	Mineral water	France	60.4	59.9	1017
*P. mandelii* subgroup	*P. farris* SWRI79	Rhizosphere of wheat	Iran	58.7	59.9	1017
	*P. migulae* NBRC 103157	Mineral water	France	59.1	59.7	1017
*P. fluorescens* subgroup	*P. grimontii* DSM 17515	Mineral water	France	60.1	59.4	1017
	*P. marginalis* ICMP 3553	*Cichorium intybus* leaf	USA	60.4	58.1	1017
	*P. palleroniana* CCUG 51524	*Oryza sativa*	Cameroon	60.5	58.0	1017
	*P. panacis* DSM 18529	Ginseng root lesions	South Korea	61.1	59.4	1017
*P. massiliensis* group	*P. typographi* CA3A	European Bark Beetle (*Ips typographus*)	Czech Republic	62.1	61.7	1017
*P. anguilliseptica* group (High GC%)	*P. benzenivorans* DSM 8628	Soil	USA	65.2	55.4	1014
*P. oryzihabitans* group (High GC%)	*P. oryzihabitans* DSM 6835	Rice paddy	Japan	66.2	67.0	1014
	*P. psychrotolerans* DSM 15758	Water	Austria	65.3	66.2	1014
	*P. rhizoryzae* RY24	Rice seeds	China	64.8	65.1	1014

The study of the prevalence of *AcdS* in *Pseudomonas* type strains was conducted by BLASTp (standard parameters) in the NCBI database, using the *Pseudomonas* sp. UW4 functional *AcdS* protein sequence (WP_015096487.1) as query [65]. Positive hits were considered for values of identity > 50%.

**Table 2:** Properties of the *Pseudomonas* sp. UW4 and other studied bacterial ACC deaminase enzymes.

Strain	K_M_ (mM)	k_cat_ (min^−1^)	pH Optimum	Temperature Optimum (°C)	Structure and Molecular Mass (KDa)	Reference
*Pseudomonas* sp. UW4	3.4 ± 0.2	146 ± 5	8.0	37	Homotetramer 168 kDa	[65]
*Methylobacterium nodulans* ORS2060	0.8 ± 0.04	111.8 ± 0.2	8.0	50	Homotetramer 144 kDa	[71]
*Methylobacterium radiotolerans* JCM2831	1.8 ± 0.3	65.8 ± 2.8	8.0	45	Homotetramer 144 kDa	[71]
*Amycolatopsis methanolica* 239	1.7 ± 0.2	5.1 ± 0.2	8.5	60	Homotetramer 144 kDa	[72]

**Table 3:** Studies on the role of ACC deaminase (*acdS* gene expression) in different *Pseudomonas* strains.

*Pseudomonas* Strain	Effects of acdS Deletion	Reference
*Pseudomonas* sp. GR12-2	▪ Unable to promote canola root elongation	[35]
*Pseudomonas* sp. UW4	▪ Unable to promote canola root elongation ▪ Decreased ability to protect canola, cucumber, and tomato from salt stress ▪ Decreased ability to reduce cadmium accumulation in several plants ▪ Unable to promote the colonization process of mycorrhiza ▪ Decreased ability to protect tomato from *Agrobacterium* infection ▪ Decreased ability to promote pine growth and protect it from nematode infection	[60,61,63,78,79,80,81]
*P. brassicacearum* Yss6	▪ Lost the ability to decrease flower senescence ▪ Decreased ability to promote tomato growth and protect it from salt stress ▪ Lost the ability to promote the nodulation process of alpha and beta-rhizobia	[17,77,82]
*P. migulae* 8R6	▪ Lost the ability to decrease flower senescence ▪ Decreased ability to promote tomato growth and protect it from salt stress ▪ Decreased ability to protect periwinkle from phytoplasma infection	[17,62]
*P. ogarae* F113	▪ Lost the ability to promote maize root growth and seed germination	[75,83]
*P. palleroniana* Q1	▪ Decreased ability to promote the nodulation process of rhizobia	[84]
*P. azotoformans* CHB 1107	▪ Decreased ability to promote tomato plant growth and resistance to salt stress	[85]
	Effects of Exogenous *acdS* Expression	
*P protegens* CHA0	▪ Gained the ability to promote canola root elongation ▪ Improved its ability to protect cucumber against Pythium damping-off, and potato tubers against *Erwinia* soft rot	[76]
*P. putida* ATCC 17399	▪ Increased plant growth promotion activities (shoot, root) ▪ Increased ability to protect tomato plants from flooding stress	[50]
*P. frederiksbergensis* OS211	▪ Increased plant growth promotion activities ▪ Increased ability to protect tomato plants from chilling stress	[86]

## Data Availability

Not applicable.
